# Garlic-braid–derived activated biochar as a high-performance sulfur host for lithium–sulfur batteries

**DOI:** 10.3389/fchem.2026.1860681

**Published:** 2026-06-11

**Authors:** Lucía del Carmen Navarro Di Mari, Francisco J. García-Soriano, Flavia Lobo Maza, Fernando Cometto, Guillermina L. Luque, Sofía Raviolo, María Victoria Bracamonte

**Affiliations:** 1 Centro de Investigaciones Fisicoquímicas, Teóricas y Aplicadas, (CIFTA, CREAS-FACEN, UNCA), Catamarca, Argentina; 2 Department of Materials Chemistry, Kemijski Institute, Ljubljana, Slovenia; 3 Instituto de Investigaciones en Físico-Química de Córdoba (INFIQC, FCQ – UNC), Córdoba, Argentina; 4 Departamento de Fisicoquímica, Facultad de Ciencias Químicas, Universidad Nacional de Córdoba, Córdoba, Argentina; 5 Departamento de Química Teórica y Computacional, Facultad de Ciencias Químicas, Universidad Nacional de Córdoba, Córdoba, Argentina; 6 Instituto de Física Enrique Gaviola (IFEG, FaMAF - UNC), Córdoba, Argentina

**Keywords:** biochar, cathode, garlic braid, hierarchical structure, sulfur batteries

## Abstract

Lithium-sulfur (Li-S) batteries are promising next-generation energy storage systems due to their high theoretical energy density. However, their practical implementation is hindered by the low conductivity of sulfur and the polysulfide shuttle effect. In this work, garlic-braid-derived biochar was investigated as a sustainable sulfur host for Li-S battery cathodes. Biochar obtained from garlic braid biomass was chemically activated using potassium hydroxide to generate a highly porous carbon matrix. Sulfur was subsequently infiltrated into the activated biochar, and the resulting composite was evaluated as a cathode material in Li-S batteries. The activated biochar exhibited a well-developed porous structure, enabling a sulfur infiltration degree of 48 wt.%. The optimized cathode delivered an initial discharge capacity of 1,015 mAh/g and stabilized at approximately 700 mAh/g after 80 cycles at 0.1 C. Furthermore, a specific capacity of 491 mAh/g was achieved at 1 C, demonstrating favorable rate capability. The cells also exhibited stable Coulombic efficiency and good capacity retention during prolonged cycling. The enhanced electrochemical performance is attributed to the hierarchical porous structure generated by chemical activation, which promotes sulfur confinement, facilitates ion transport, and improves sulfur utilization. These findings demonstrate that garlic-braid-derived activated biochar is a promising and sustainable sulfur host for Li-S batteries, highlighting the importance of both biomass selection and pore structure engineering in the development of high-performance biochar-based cathodes.

## 1 Introduction

The growing demand for high-energy-density storage systems has intensified interest in beyond lithium-ion technologies. Lithium-sulfur batteries (LSBs) are among the most promising candidates, offering a theoretical specific capacity of 1,675 mAh/g and an energy density of 2,600 Wh/kg, while relying on sulfur—an abundant, low-cost, and environmentally benign active material (Aifantis et al., 2022; Nagde and Dhoble, 2021). However, several fundamental challenges continue to hinder their commercialization. The dissolution of intermediate lithium polysulfides (Li_2_S_x_, 4 ≤ x ≤ 8) into the electrolyte and their subsequent shuttling between electrodes leads to irreversible active material loss, rapid capacity fade, and poor Coulombic efficiency (Jyothirmai and Ravva, 2022; Prehal et al., 2021; Prehal et al., 2022). Further complications arise from the low electronic conductivity of sulfur and its discharge products, the large volumetric expansion (∼80%) during lithiation, and safety concerns associated with lithium metal anodes (Li and Sun, 2018; Xiong et al., 2019). Various strategies have been proposed to enhance LSB performance, including modifying the separator (Yin et al., 2018), adjusting the electrolyte composition (Lin et al., 2021), protecting the anode electrode (Calderón et al., 2020), among others. Engineering the cathode host material has proven to be one of the most effective strategies to mitigate these issues. Porous carbon hosts can physically confine sulfur within their pore network, limit polysulfide dissolution, and provide the electronic conductivity necessary for efficient electrochemical utilization of the active material (Li et al., 2018; Ogoke et al., 2017). The effectiveness of this approach depends critically on the pore structure of the host: a high surface area and well-developed microporosity favour strong sulfur confinement and high loading, while the presence of interconnected mesopores and macropores facilitates electrolyte penetration and ion transport during cycling (Wang et al., 2019).

Beyond electrochemical performance, the sustainability profile of the synthesis route is a critical consideration in the development of next-generation battery materials. Green chemistry principles advocate for the use of renewable feedstocks, minimal reagent loading, and processes that avoid hazardous intermediates, criteria that biomass-derived activated carbons are well positioned to meet (Reis et al., 2020; Wang and Kaskel, 2012). In this context, the choice of precursor, the activation protocol, and the overall carbon yield from raw biomass collectively determine the environmental footprint of the cathode host (Reis et al., 2020). Agricultural residues are particularly attractive in this regard, as they convert a waste management burden into a value-added material without competing with food supply chains (Qiu et al., 2025). KOH chemical activation, while requiring an alkaline reagent, can be performed at relatively low impregnation ratios and yields a carbon framework that is subsequently purified by acid washing and water rinsing, steps that are compatible with standard laboratory and industrial workflows (Raviolo et al., 2025; Wang and Kaskel, 2012). Crucially, the carbon yield and reagent efficiency of the activation process directly influence the scalability and practical viability of the resulting material.

Activated biochars derived from biomass waste represent a particularly attractive class of carbon hosts for LSBs. They can be produced from renewable, low-cost precursors, and their porosity and surface chemistry can be tuned through the choice of activation conditions. KOH chemical activation at high temperatures is especially effective, promoting the development of extensive microporous networks alongside partial graphitization of the carbon framework, which enhances both the pore volume available for sulfur loading and the electronic conductivity of the host (Kwon et al., 2021; McNulty et al., 2020). A variety of biomass precursors have been explored in literature, including olive pomace (Ga et al., 2025), yerba mate waste (Tesio et al., 2023), and spent brewer’s grain (Raviolo et al., 2025), demonstrating that competitive electrochemical performance can be achieved from agricultural residues.

Garlic is produced globally on a large scale, and its commercialization generates significant quantities of braid waste—the dried stems and leaves used to bundle bulbs—that are typically discarded (Jain et al., 2025; Qiu et al., 2025). This lignocellulosic residue has not previously been investigated as a sulfur host for LSBs, representing an unexplored opportunity to add value to an abundant agricultural byproduct. In this work, we report the synthesis of a KOH-activated biochar derived from garlic braid waste and its application as sulfur host in the cathode of lithium-sulfur cells. The optimized composite achieves a sulfur loading of 48 wt.%, delivers an initial discharge capacity of 1,015 mAh/g stabilizing at approximately 700 mAh/g over extended cycling at 0.1 C, and maintains a capacity of 491 mAh/g at 1 C, demonstrating the suitability of this waste-derived carbon as a high-performance sulfur host for sustainable battery applications.

## 2 Materials and methods

### 2.1 Synthesis and sulfur infiltration of activated biochar

Garlic braids were obtained from retail vegetable outlets supplied by farmers’ markets in Córdoba Province, Argentina. The biomass was milled to reduce its particle size using a Labklass propeller grain mill model hc-100 at 28,000 rpm for 3 min at 850 W of power. After that, it was carbonized under a nitrogen atmosphere in a tubular furnace (GSL-1100), at 500 °C for 30 min, with a heating rate of 5 °C/min. This material was labelled as G5. The carbon yield from G5 biochar was 32.5%. A subset of G5 underwent chemical activation using potassium hydroxide (KOH). These powders (1:1 mass ratio) were mixed at 400 rpm for 10 min using a Fritsch ball mill (Model Pulverisette 7). Then, pyrolyzed at 900 °C for 1 h under a nitrogen atmosphere. After cooling, the resultant material was thoroughly washed first with a 7 wt.% solution of hydrochloric acid (HCl) and, then deionized water until reaching a neutral pH. The obtained biochar was labelled as G5A9. This biochar was then infiltrated with sulfur as follows: G5A9 was mixed with elemental sulfur (Sigma-Aldrich) at a 3:7 mass ratio in an agate mortar. The mixture was then heated under vacuum to 155 °C for 5 h at a rate of 5 °C/min, followed by a step at 300 °C for 30 min to remove residual sulfur from the external surface. The sulfur-infiltrated sample was labelled G5A9+S.

### 2.2 Physicochemical characterization

To determine the surface area and pore volume of the biochar, nitrogen gas adsorption-desorption isotherms were performed at 77 K using a Micromeritics ASAP 2020 instrument. The total surface area and pore volume were calculated using the Brunauer-Emmett-Teller (BET) equation and Gurvich’s rule, respectively. The α-plot method was used to determine the micropore volume and mesopore area. The pore size distribution of the sample (PSD) was determined by applying the Quenched-Solid Density Functional Theory (QSDFT) model for slit/cylindrical pores in the adsorption branch. Crystal structure was evaluated by X-ray diffraction (XRD) using a Panalytical X’Pert Pro diffractometer with Cu Kα radiation (λ = 1.541 Å). Measurements were recorded from 10° to 80° 2θ at a scan rate of 6°/min, under 40 kV and 30 mA conditions. To visualize the morphology of the samples, Field-Emission Scanning Electron Microscopy (FE-SEM) (Zeiss) was used with an acceleration energy of 3 keV. The chemical composition of the samples was determined by Energy-Dispersive X-ray Spectroscopy (EDS) attached to the FE-SEM, operating at 20 keV. X-ray Photoelectron Spectroscopy (XPS) measurements were conducted using a Thermo Fisher Scientific K Alpha XPS system using non monochromatized Al-Kɑ 1200W. Thermo Gravimetric Analysis (TGA) was performed on a TA Instrument Corporation Q500, utilizing a nitrogen atmosphere and a heating rate of 10 °C/min with a maximum temperature of 600 °C.

### 2.3 Electrochemical characterization

Electrodes were fabricated by mixing 80 wt.% of the G5A9+S composite, 10 wt.% of conductive carbon (Ketjen Black), and 10 wt.% of polyvinylidene fluoride (PVdF), using N-methyl-2-pyrrolidone (NMP) as solvent. The mixture was ball-milled at 600 rpm for 10 min in a Planetary Fritsch ball mill (Model Pulverisette 7). The resultant slurry was used to coat a brushed aluminium foil using the doctor blade method and dried at 80 °C for 2 h. The electrodes, with an average mass loading of 1.5 mgs/cm^2^ (1.13 cm^2^ electrode area), were used to assemble CR2032 coin cells with lithium foil as a counter electrode. These cells were assembled in an argon-filled glovebox using lithium foil (disk of 12 mm diameter) as anode and reference, Celgard 2325 as separator, and 20 µL/mg_S_ of 1 M lithium bis(trifluoromethane sulfonimide) (LiTFSI) in a 1:1 (v/v) mixture of 1,3-dioxolane (DOL) and 1,2-dimethoxyethane (DME) as electrolyte. The electrochemical performance was analysed in a potential window of 2.8 V and 1.8 V vs. Li^+^/Li at room temperature. The galvanostatic cycling was evaluated at 0.1 C, being C 1675 mAh/g. Cyclic voltammograms (CV) were performed at different scan rates from 0.1 mV/s to 1 mV/s. C-rate tests were performed changing the current rates every 10 cycles from: 0.1 C, 0.3 C, 0.5 C, 1 C, 2 C, 0.5 C, and 0.1 C. The Galvanostatic Intermittent Titration Technique (GITT) was performed with the following steps: after a first formation, a cycle current pulse of C/10 for 15 min was applied, followed by a relaxation time of 4 h. All tests were conducted at room temperature. The GITT and CV experiments were performed using a Biologic Science Instrument Model BCS-810, while galvanostatic discharge/charge C-rate testing was done with a Landt CT2001 Series Battery Test System.

## 3 Results and discussion

### 3.1 Physicochemical characterization of biochar

**FIGURE 1:**
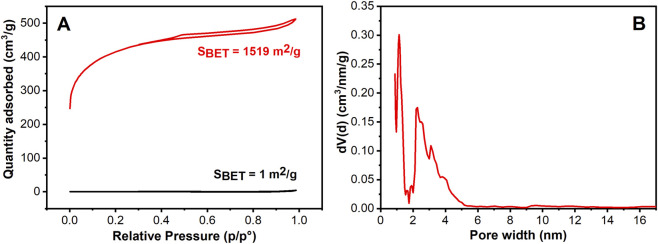
N_2_ adsorption–desorption isotherms of G5 (black) and G5A9 (red) **(A)**, and pore size distribution of G5A9 **(B)**. Surface area, pore volume, and the micro-/mesopore contributions for both samples are summarized in Table 1.

N_2_ adsorption/desorption isotherms were acquired for the samples before and after activation (Figure 1A). The total surface area, pore volume, and the contribution of micro- and mesopores for each sample are summarized in Table 1.

**TABLE 1:** Textural parameters of the G5 and G5A9 samples derived from N_2_ adsorption-desorption isotherms.

Sample	S_BET_	S_meso_	S_micro_	V_T_	V_meso_	V_micro_
	(m^2^/g)			(cm^3^/g)		
G5	1	-	-	0.01	-	-
G5A9	1,519	324	1,195	0.72	0.29	0.43

As shown in Figure 1A, G5 exhibits negligible N_2_ adsorption, consistent with its low surface area and pore volume (Table 1), indicating a non-porous structure. In contrast, G5A9 displays a Type Ib isotherm, typical of microporous carbons (Thommes et al., 2015), with a steep uptake at low relative pressures (p/p^0^) associated with micropore filling. This behavior suggests a pore structure predominantly composed of micropores with a broad size distribution, along with a minor contribution from narrow mesopores. The appearance of an H4-type hysteresis loop above p/p^0^ ≈ 0.4 indicates the presence of slit-like pores, while the gradual increase in adsorption at higher relative pressures points to additional mesoporosity. As shown in Figure 1B, the pore size distribution of G5A9 is quasi-bimodal, with a main peak at ∼1.1 nm and a broader contribution between 2 and 5 nm. This pore structure leads to a high S_BET_ of 1,519 m^2^/g and a total pore volume of 0.72 cm^3^/g, with micropores contributing ∼80% of the surface area and ∼60% of the pore volume (Table 1). Overall, activation results in a well-developed micro–mesoporous network and a substantial increase in accessible surface area. This hierarchical porous structure would be directly linked to the improved electrochemical performance of the G5A9+S cathode. Micropores (<2 nm) are expected to promote strong physical confinement of sulfur species and soluble polysulfides, suppressing shuttle effects and enhancing sulfur utilization. The material’s mesopores (2–5 nm) facilitate electrolyte penetration, improve Li^+^ transport, and provide ion diffusion pathways while accommodating volume changes associated with sulfur conversion. In addition, larger interparticle voids and macroporous transport channels generated by the carbon natural framework can act as electrolyte reservoirs and reduce mass-transport limitations at higher sulfur loading. Therefore, the synergistic contribution of micro-, meso-, and transport macroporosity likely underpins the enhanced redox kinetics and cycling stability observed for G5A9+S, in agreement with recent reports emphasizing hierarchical pore architectures as key design elements for high-performance biomass-derived sulfur hosts (Wu et al., 2025; Zoubir et al., 2024).

**FIGURE 2:**
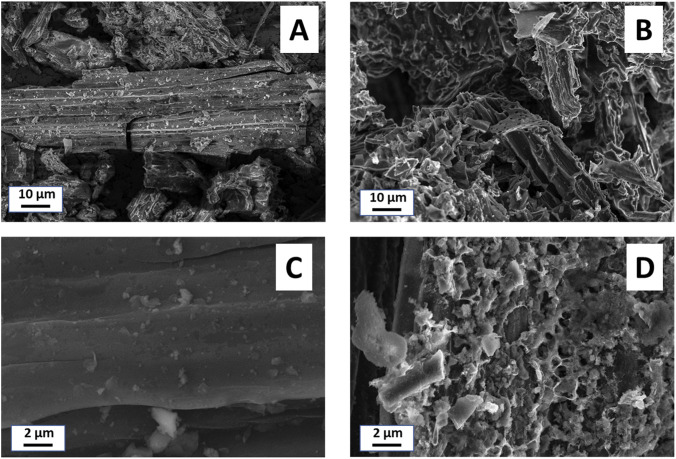
SEM images at two magnifications of G5 **(A,C)** and G5A9 **(B,D)**. Chemical activation promotes the development of an interconnected hierarchical porous structure in G5A9 compared to the denser carbon framework of G5.

The morphological evolution of the biochars was examined by SEM (Figure 2), revealing clear structural differences between the G5 and G5A9 samples. G5 (Figures 2A,C) exhibits a compact carbon framework with relatively smooth surfaces and a limited number of large voids, consistent with its low porosity. In contrast, G5A9 (Figures 2B,D) displays a markedly roughened texture with a high density of pores distributed throughout the matrix, evidencing the effectiveness of KOH activation in generating porosity. The presence of smaller pores embedded within larger cavities suggests the coexistence of meso- and macropores, leading to a hierarchical and interconnected architecture that is expected to facilitate electrolyte infiltration and ion transport (Ga et al., 2025). The macropore size distribution (Supplementary Figure S1) yields an average diameter of (0.28 ± 0.09) μm.

Complementary EDS analysis (Supplementary Figure S2) indicates that the pristine G5 contains inorganic impurities such as Ca, K, Cl, S, and P, originating from the biomass precursor. After activation and acid washing, a substantial decrease in the relative content of these elements is observed, consistent with the removal of most inorganic residues.

**FIGURE 3:**
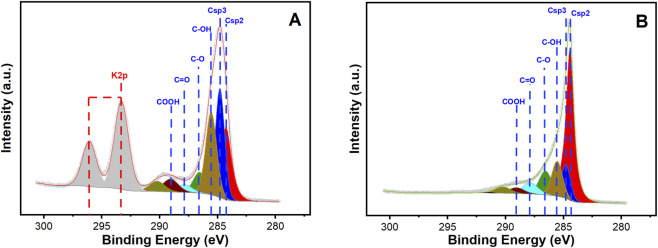
High-resolution C 1s XPS spectra of pristine G5 **(A)** and activated G5A9 **(B)**. Activation increases the sp^2^ carbon contribution and removes the K 2p signal observed in the non-activated sample.

To elucidate the chemical environment of the samples, the C 1s core-level spectra (Figure 3) were systematically analyzed. In the non-activated sample (G5, Figure 3A), a significant contribution from oxidized carbon species is observed, along with partial overlap with the K 2p doublet. In contrast, the activated samples exhibit highly similar C 1s profiles, dominated by an asymmetric peak centered at (284.4 ± 0.1) eV, characteristic of sp^2^-hybridized carbon atoms in delocalized C–C bonds, indicative of graphitic domains. Contributions within the 284.8 to ∼289 eV range arise from sp^3^-hybridized carbon species, including carbon atoms bonded to carbon and hydrogen (e.g., defective or edge sites at ∼284.8 eV) as well as carbon atoms associated with oxygen-containing functional groups.

Deconvolution of the C 1s spectra reveals six distinct components, providing further insight into the surface chemistry. In addition to the graphitic sp^2^ contribution at (284.4 ± 0.1) eV and the sp^3^ component at 284.8 eV (associated with amorphous carbon), three oxygenated functionalities are identified: C–OH at (285.6 ± 0.1) eV, C–O at (286.5 ± 0.2) eV, and carbonyl groups (C=O) at (287.9 ± 0.2) eV. A sixth contribution at (289.1 ± 0.2) eV is assigned to carboxylic acid groups (COOH), while π–π* shake-up features are also present at higher binding energies (Al-Gaashani et al., 2019; Bracamonte et al., 2023; Ga et al., 2025; Raviolo et al., 2025).

**FIGURE 4:**
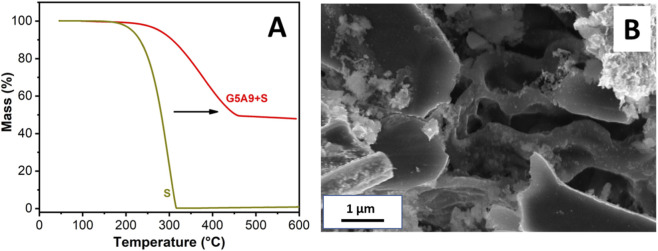
**(A)** Thermogravimetric analysis of the G5A9+S composite (red) and pristine sulfur (olive). **(B)** SEM image of G5A9+S. The composite contains approximately 48 wt.% sulfur, and the shift in sulfur evaporation to higher temperatures indicates sulfur confinement within the porous biochar host.

A marked increase in the relative intensity of the sp^2^ carbon contribution is observed for G5A9 (Figure 3B) compared to G5, consistent with the higher activation temperature (900 °C). This treatment promotes structural rearrangement within the carbon matrix, leading to enhanced ordering and partial graphitization. Accordingly, the relative contribution of sp^3^-hybridized carbon decreases in G5A9, reflecting the predominance of disordered, amorphous domains in the untreated G5 sample. This structural evolution is quantitatively supported by the increase in the C-sp^2^/sp^3^ ratio from 0.67 in G5 to 3.74 in G5A9, confirming that KOH activation at 900 °C effectively induces graphitization in the biochar. Concomitantly, potassium is efficiently removed during the transformation from G5 to G5A9, as evidenced by the disappearance of the K 2p signal in the XPS survey spectra (Supplementary Figure S3). This is accompanied by a reduction in the relative intensity of other impurity-related signals (e.g., Ca 2p and P 2p), indicating a substantial decrease in inorganic residues following activation and subsequent acid washing. Overall, these results demonstrate that the combined treatment not only enhances structural ordering but also effectively eliminates residual inorganic species, yielding a cleaner carbon matrix. After the comprehensive characterization of samples G5 and G5A9, the activated material was subsequently infiltrated with sulfur. The sulfur content was determined by thermogravimetric analysis (TGA) under N_2_ atmosphere. As shown in Figure 4A, the thermal decomposition profiles of pristine sulfur and G5A9+S exhibit markedly different behaviours. While pure sulfur displays a sharp and well-defined weight loss, the composite shows a broader and more gradual mass-loss profile, indicative of a heterogeneous sulfur distribution within the carbon matrix. The mass-loss of the G5A9+S indicates a sulfur content of 48 wt% confined within the pore network. Additionally, the composite exhibits a clear shift toward higher evaporation temperatures, compared with the pristine sulfur, with a maximum around 450 °C. This effect is further evidenced in the derivative thermogravimetric curves (Supplementary Figure S4), where the peak associated with pore-confined sulfur appears at higher temperatures compared to free sulfur, indicating stronger confinement and enhanced interactions between sulfur and the carbon framework (Bracamonte et al., 2023; Garcia-Soriano et al., 2020; Raviolo et al., 2023).

Additional X-ray diffraction analysis of G5A9 and sulfur-loaded G5A9+S (Supplementary Figure S5) further supports the classification of the material as an infiltrated activated biochar. Both samples exhibit broad diffuse features centered near 2θ ≈ 24°–26° and around 43°–45°, corresponding to the (002) and (100) reflections of carbon, characteristic of poorly graphitized biomass-derived biochars rather than highly ordered graphitic porous carbons (Rigollet et al., 2025; Sun et al., 2025). The absence of sharp graphitic reflections confirms the predominantly disordered carbon framework. After sulfur infiltration, the preservation of these broad carbon reflections indicates that the host structure remains intact, while subtle changes in intensity and background are consistent with sulfur incorporation within the porous network rather than structural collapse (Shinkarova et al., 2025).

Complementary N_2_ adsorption–desorption measurements (Supplementary Figure S6) further confirm the successful infiltration of sulfur into the porous network. Compared to pristine G5A9 (Figure 1; Table 1), the total pore volume decreases drastically from 0.72 cm^3^/g to 0.002 cm^3^/g, while S_BET_ is reduced from 1,519 to 0.5 m^2^/g. This near-complete loss of accessible porosity indicates extensive occupation of both micro- and mesopores by sulfur, consistent with TGA results and supporting efficient sulfur confinement within the hierarchical carbon host. Morphological and compositional analyses provide additional evidence of sulfur incorporation. SEM images of G5A9+S (Figure 4B) reveal that the porous structure is largely preserved after infiltration, although pore openings appear partially occluded. Corresponding EDS elemental mapping (Supplementary Figure S7) demonstrates a homogeneous distribution of sulfur throughout the carbon matrix, confirming effective impregnation without the formation of large, segregated sulfur domains. These findings show that KOH activation transforms this otherwise discarded biomass into a hierarchically porous carbon with a markedly increased C-*sp* ^*2*^ */sp* ^*3*^ ratio from 0.67 to 3.74, confirming effective graphitization alongside the development of a high surface area microporous network. The use of a mild KOH-to-biochar mass ratio of 1:1, among the lowest reported for chemically activated biomass-derived sulfur hosts (Aguirre et al., 2025; Grace and Martha, 2026; Lee et al., 2021; Spyrou et al., 2022; Tesio et al., 2023; Xia et al., 2020), further underscores the resource efficiency of the process, minimizing reagent consumption while achieving competitive textural properties. After sulfur infiltration (48 wt.%), the G5A9+S composite exhibits near-complete pore filling and homogeneous sulfur distribution, confirming the suitability of this waste-derived carbon as a functional cathode host. The electrochemical performance of G5A9+S as a cathode for Li-S batteries is explored in the following section.

### 3.2 Electrochemical characterization

**FIGURE 5:**
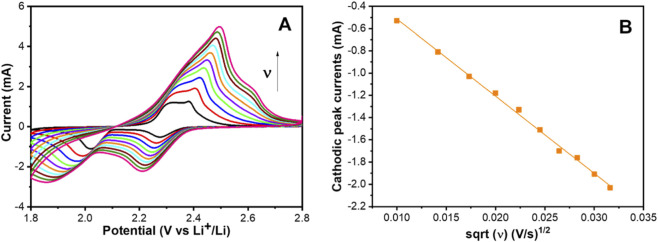
**(A)** Cyclic voltammograms of the G5A9+S cell at scan rates from 0.1 to 1.0 mV s^-1^. **(B)** Cathodic peak current as a function of the square root of scan rate. Scan rates were increased in increments of 0.1 mV s^-1^.

The electrochemical performance of the sulfur-infiltrated material was evaluated using complementary techniques. Cyclic Voltammetry (CV) were used to investigate redox processes and kinetic evolution. Figure 5A presents the CV profiles, where two cathodic peaks at 2.20 V and 1.90 V vs. Li^+^/Li are attributed to the stepwise reduction of elemental sulfur (S_8_) to soluble long-chain lithium polysulfides (Li_2_S_x_, 4 ≤ x ≤ 8), followed by their further conversion to insoluble short-chain species (Li_2_S_2_/Li_2_S). The anodic peaks at 2.30 V and 2.40 V correspond to the reverse oxidation process, involving the transformation of Li_2_S_2_/Li_2_S back to higher-order polysulfides and ultimately to elemental sulfur (Prehal et al., 2022; Salvo et al., 2024; Zhang et al., 2021).

A systematic shift of both cathodic and anodic peaks with increasing scan rate is observed, indicating polarization effects associated with kinetically controlled electrochemical processes (Bard and Faulkner, 2025). The apparent lithium-ion diffusion coefficient (D_app_) was estimated using the Randles–Ševčík equation (Supplementary Note S1). From the linear fit of the peak current of the first cathodic peak at ∼2.2 V vs. the square root of the scan rate (Figure 5B), a D_app_ value of 4.2 × 10^−9^ cm^2^/s was obtained. This value falls within the typical range reported for lithium–sulfur systems, confirming reasonably fast redox kinetics in the G5A9+S electrode (Bracamonte et al., 2023).

**FIGURE 6:**
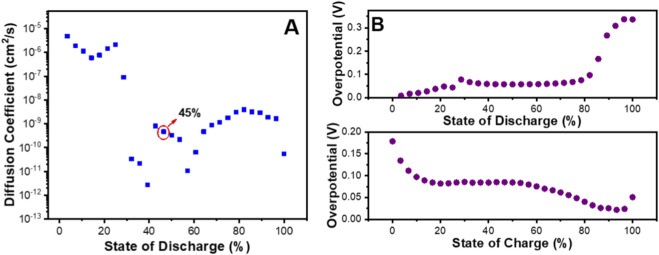
**(A)** Lithium diffusion coefficient as a function of state of discharge (SoD). **(B)** Evolution of overpotential as a function of SoD (upper panel) and state of charge (SoC, lower panel), obtained from GITT measurements of the G5A9+S cathode.

To gain deeper insight into lithium-ion transport kinetics, the apparent diffusion coefficient (D_app_) was further evaluated using galvanostatic intermittent titration technique (GITT) measurements (see Supplementary Note S2; Supplementary Figure S8). The D_app_ values extracted from the GITT response (Supplementary Equation SE4) as a function of the state of discharge (SoD) are presented in Figure 6A. At intermediate discharge (≈45% SoD), corresponding to potentials in the range of ∼2.3–2.4 V associated with the reduction of long-chain polysulfides, a relatively high diffusion coefficient of 1.44 × 10^−6^ cm^2^/s is obtained. As the discharge proceeds toward lower potentials (∼2.0–2.1 V), where the conversion to Li_2_S_2_/Li_2_S takes place, D_app_ decreases to 8.85 × 10^−8^ cm^2^/s, reflecting the sluggish kinetics and solid-phase transformation characteristic of this stage. This trend is consistent with the CV analysis, where the diffusion coefficients derived from peak currents fall within the same order of magnitude, supporting the reliability of the kinetic assessment (Bracamonte et al., 2023; Garcia-Soriano et al., 2020).

The evolution of overpotential during discharge (Figure 6B, upper panel) provides mechanistic insight into the rate-limiting steps governing sulfur redox. In the initial stage (0%–10% SoD), the low overpotential (0.01–0.06 V) reflects fast kinetics associated with the solid–liquid transition from S_8_ to soluble long-chain polysulfides, where charge transfer is not rate-limiting and transport occurs predominantly in the electrolyte phase. Between 10% and 60% SoD, the stabilization of the overpotential at ∼0.075 V indicates a quasi-steady-state regime in which the rate of polysulfide conversion is balanced by their diffusion within the porous network. In this regime, mesopores act as ion-transport channels, minimizing concentration gradients and sustaining reaction uniformity. At higher SoD (>80%), the sharp increase in overpotential arises from the nucleation and growth of insulating Li_2_S_2_/Li_2_S phases, which progressively block active sites and increase both charge-transfer resistance and ionic transport limitations within micropores, where confinement effects become dominant. During charge (Figure 6B, lower panel), the mirrored overpotential profile as a function of SoC reflects the reverse sequence of reactions. The comparable magnitude of overpotentials in charge and discharge indicates that the deposition/dissolution of Li_2_S remains largely reversible, suggesting that the porous carbon matrix effectively mitigates irreversible passivation and maintains accessible reaction interfaces.

**FIGURE 7:**
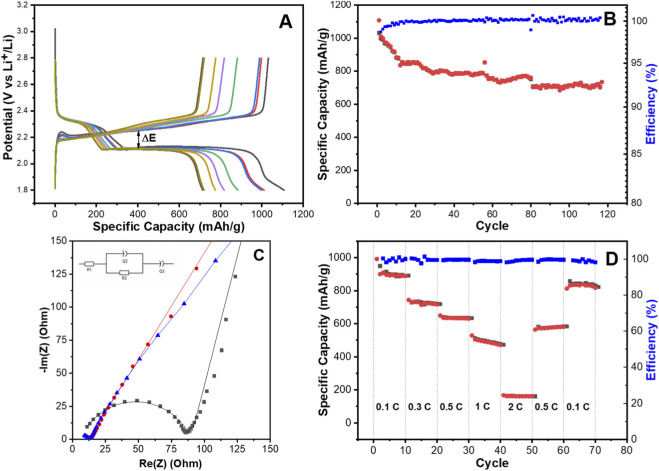
Electrochemical performance of the G5A9+S cathode: **(A)** galvanostatic voltage profiles at 0.1 C; **(B)** specific capacity versus cycle number; **(C)** Nyquist plot of electrodes in the fully lithiated state, for the 5th (red) and 10th cycles (blue). For comparison, the Nyquist plot of pre-cycled electrodes is included (black). The measured data (symbol) are presented together with the corresponding fitted curves (solid line). **(D)** Specific capacity and Coulombic efficiency at different C-rates.

The electrochemical performance of the G5A9+S cathode was further evaluated by galvanostatic charge–discharge measurements. The voltage profiles at different cycles (Figure 7A) exhibit the typical features of lithium-sulfur chemistry, with two well-defined discharge plateaus and a single charge plateau (Huan et al., 2018; Prehal et al., 2022). The high-voltage plateau at ∼2.4 V is associated with the reduction of elemental sulfur (S_8_) to long-chain soluble polysulfides (Li_2_S_x_, 4 ≤ x ≤ 8), followed by a sloping region down to ∼2.1 V corresponding to their progressive conversion into short-chain species. The low-voltage plateau at ∼2.1 V reflects the formation of insoluble Li_2_S_2_/Li_2_S (Prehal et al., 2022; Salvo et al., 2024; Zhang et al., 2021). The relatively stable plateau positions upon cycling indicate that polarization does not drastically increase, consistent with maintained ionic and electronic pathways within the electrode.

The evolution of the overpotential during cycling was assessed by tracking the potential hysteresis (ΔE) at a fixed specific capacity of 400 mAh/g (Supplementary Figure S9). A gradual increase in ΔE is observed upon cycling, indicating enhanced polarization effects. This behavior is commonly attributed to increased interfacial resistance arising from the accumulation of insulating Li_2_S_2_/Li_2_S species at the cathode, parasitic reactions at the anode due to dissolved polysulfides, and changes in electrolyte resistance associated with continuous polysulfide shuttling (Conder et al., 2017; García-Soriano et al., 2020).

The cycling stability and Coulombic efficiency are presented in Figure 7B. The G5A9+S electrode delivers an initial discharge capacity of 1,015 mAh/g, followed by a moderate decay during the initial cycles and subsequent stabilization at ∼700 mAh/g from cycle 15 onwards. The Coulombic efficiency rapidly approaches ∼99% after the initial formation cycles, while the lower efficiency in the first cycle (97%) is attributed to SEI/CEI formation and irreversible processes (Tarimo et al., 2025).

Further insight into the evolution of interfacial properties was obtained by electrochemical impedance spectroscopy (EIS) in the fully lithiated and fully delithiated states, as shown in Figure 7C; Supplementary Figure S10, respectively. The Nyquist plots display a depressed semicircle in the high-to-medium frequency region, associated with charge-transfer resistance, followed by a low-frequency Warburg-type response related to diffusion processes. To quantitatively analyse these features, the spectra were fitted using the equivalent circuit shown as an inset in Figure 7C; Supplementary Figure S9. Notably, a decrease in the charge-transfer resistance is observed upon cycling (from ∼80 Ω in cycle 1–5 Ω in cycle 10), indicating improved electrode/electrolyte interfacial properties and enhanced polysulfide adsorption within the porous carbon matrix. This behaviour is consistent with the hierarchical structure of the biochar, where micropores promote sulfur confinement while mesopores facilitate ion transport, ultimately improving reaction kinetics, in line with previous reports. (Drvaric Talian et al., 2022; Grace and Martha, 2026; Waluś et al., 2020). The rate capability of the G5A9+S cathode was evaluated at increasing current densities (Figure 7D). The electrode delivers average specific capacities of 888, 725, 634, 491, and 160 mAh/g at 0.1  C, 0.3  C, 0.5  C, 1  C, and 2  C, respectively. Importantly, upon returning to lower current densities, the capacity recovers to 574 mAh/g at 0.5  C and 828 mAh/g at 0.1  C, indicating good structural stability and reversibility. The corresponding charge–discharge profiles at different rates (Supplementary Figure S11A) show increasing overpotential with current density, as confirmed by the progressive increase in ΔE (Supplementary Figure S11B). This effect arises from kinetic and mass transport limitations at high rates. Notably, ΔE decreases again upon returning to lower C-rates, further confirming the reversibility of the electrode and the absence of significant degradation during high-rate operation (Bracamonte et al., 2023; Kaciulis, 2012).

To contextualize the electrochemical performance of the G5A9+S cathode, a comparison with previously reported biochar-based sulfur hosts is presented in Table 2. The results highlight that, although the specific surface area of G5A9 (1,519 m^2^/g) is lower than that of some highly activated porous carbons and charcoals reported in the literature (>2,000 m^2^/g) (Chen et al., 2024; Sam et al., 2024; Wu et al., 2025), it lies within the range commonly associated with high-performing biomass-derived sulfur hosts. Importantly, these comparisons also show that electrochemical performance does not scale simply with maximum surface area; rather, hierarchical pore accessibility, sulfur loading, and the balance between micro- and mesoporosity strongly influence sulfur utilization and cycling stability. In this context, G5A9+S delivers a competitive specific capacity of 780 mAh g^-1^ at 0.1 C with a sulfur content of 48 wt.%, underscoring that optimized pore architecture can be as important as very high surface area alone.

**TABLE 2:** Summaries of the types of biomasses, surface areas, specific capacity at 50 cycles, Sulfur mass percentage and activation process of different works.

Biomass	Surface area (m^2^/g)	Specific capacity at cycle 50th (mAh/g)	Sulfur loading %	Activation process	Ref
Cauliflower stems	12.2	500 at 300 mA/g	60%	H_2_SO_4_ (120 C) calcination 750 C	Grace and Martha (2026)
Posidonia oceánica	1,264	441 at 0.1 C	70%	350 C and 800 C KOH 1:2	Spyrou et al. (2022)
Folium cycas	2,500	∼700 at 0.5 C	24.8%	400 C KOH 1:3 C and 800 C	Xia et al. (2020)
Walnut shell	959	250 at 0.06 C	70%	H_3_PO_4_ aq. 650 C	Aguirre et al. (2025)
Yerba mate	361	750 at 1 C	70%	800 °C without activation process	Tesio et al. (2023)
Garlic peel	2,290	∼700 at 0.5 C	78.8%	500 C + 850 C KOH (aq 45%) 1:4	Lee et al. (2021)
Garlic Braid	1,519	780 at 0.1 C	48%	500 C and 900 °C KOH 1:1	This work

Notably, several reported systems with higher surface areas and/or sulfur loadings exhibit comparable or even lower capacities, at similar or higher current densities. This indicates that electrochemical performance is not solely governed by surface area, but rather by the effectiveness of the pore architecture in promoting sulfur confinement and facilitating ion/electron transport. In this regard, the micro–mesoporous structure developed in G5A9 appears to provide an optimal balance between sulfur hosting and transport pathways. Furthermore, unlike systems requiring more aggressive or multi-step activation protocols (e.g., higher KOH ratios or combined chemical treatments), the G5A9 material is obtained through a relatively straightforward activation process (KOH 1:1, 900 °C), which enhances its scalability and practical relevance. Overall, our approach not only minimizes processing demands, but also reduces waste generation achieving competitive material properties while promoting sustainability.

## 4 Conclusion

In summary, this work demonstrates the successful valorization of garlic braid agricultural waste as a sustainable precursor for the synthesis of a high-performance porous carbon cathode host for lithium-sulfur batteries. The comprehensive characterization of G5 and G5A9 confirms that KOH chemical activation at 900 °C yielded a hierarchical micro-mesoporous carbon framework with a remarkable BET surface area of 1,519 m^2^/g, a total pore volume of 0.72 cm^3^/g, and a partially graphitized carbon matrix, as confirmed by BET, SEM, and the increased C-*sp* ^*2*^ */sp* ^*3*^ ratio by XPS characterization. The melt-diffusion sulfur infiltration process resulted in an effective sulfur loading of approximately 48 wt.%, with TGA confirming strong confinement of sulfur within the pore network, evidenced by the elevated evaporation temperature relative to pure sulfur. When evaluated as a cathode material in lithium-sulfur cells, the G5A9+S composite delivered a specific capacity of 719 mAh/g after 115 cycles at 0.1 C, alongside a competitive rate capability of 491 mAh/g at 1 C and high Coulombic efficiency throughout cycling, confirming good electrochemical stability. These results highlight the potential of low-cost, biomass-derived carbons as viable sulfur hosts for cathodes in next-generation lithium-sulfur battery technology, offering a promising route toward more sustainable and high-performance energy storage systems.

## Data Availability

The raw data supporting the conclusions of this article will be made available by the authors, without undue reservation.
