# Supplementary material for: Garlic-braid–derived activated biochar as a high-performance sulfur host for lithium–sulfur batteries
Source: Front Chem. 2026 Jun 11;14:1860681. doi: 10.3389/fchem.2026.1860681 (PMC13294861; doi:10.3389/fchem.2026.1860681)
Supplement: Supplementary file 1 [file DataSheet1.docx]

**Garlic-Braid–Derived Activated Biochar as a High-Performance Sulfur Host for Lithium–Sulfur Batteries**

**Lucía del Carmen Navarro Di Mari^1^, Francisco J. García-Soriano^2^, Flavia Lobo Maza^1^, Fernando Cometto^3,4^, Guillermina Luque^3,5^, Sofía Raviolo^6^, María Victoria Bracamonte^4,6^**

^1^Centro de Investigaciones Fisicoquímicas, Teóricas y Aplicadas, (CIFTA, CREAS-FACEN, UNCA), Av. Belgrano 300, 4700 Catamarca, Argentina

^2^Kemijski Institute, Hajdrihova ulica 19, 1000 Ljubljana, Slovenia.

^3^Instituto de Investigaciones en Físico-Química de Córdoba (INFIQC, FCQ – UNC), Haya de la Torre esq. Medina Allende, Ciudad Universitaria, 5000 Córdoba, Argentina.

^4^Departamento de Fisicoquímica, Facultad de Ciencias Químicas, Universidad Nacional de Córdoba. Haya de la Torre esq. Medina Allende, Ciudad Universitaria, 5000 Córdoba, Argentina.

^5^Departamento de Química Teórica y Computacional, Facultad de Ciencias Químicas, Universidad Nacional de Córdoba. Haya de la Torre esq. Medina Allende, Ciudad Universitaria, 5000 Córdoba, Argentina.

^6^Instituto de Física Enrique Gaviola (IFEG, FaMAF - UNC), Av. Medina Allende, Ciudad Universitaria, 5000 Córdoba, Argentina.


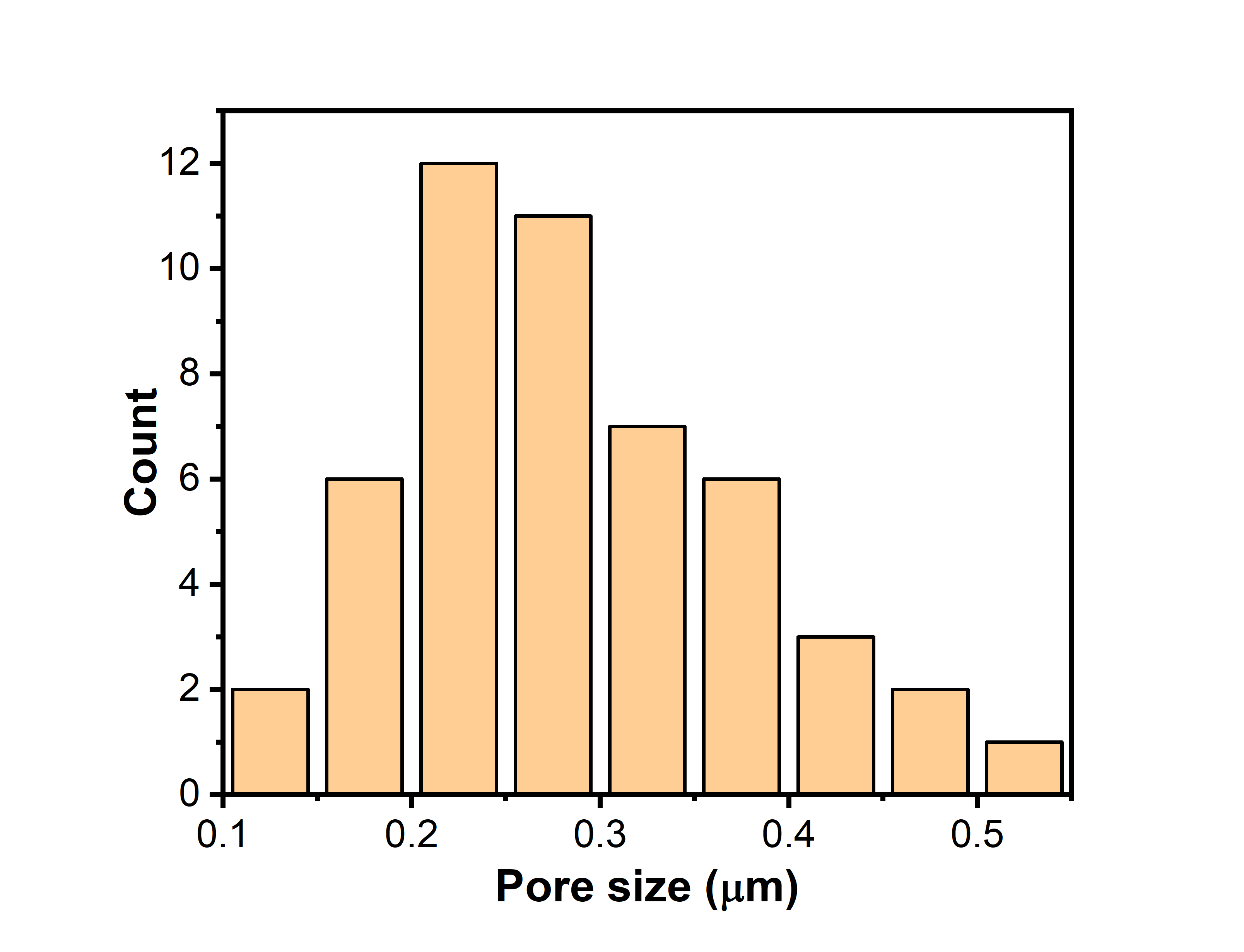


**Figure S1** - Macropores distribution for G5A9.


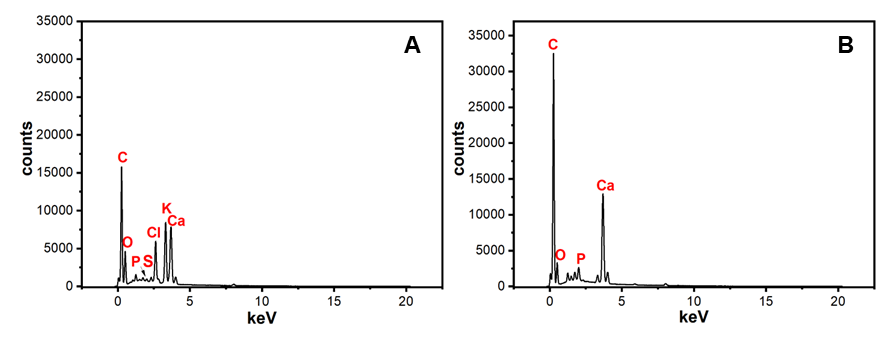


**Figure S2** – EDS spectra for **(A)** G5 and **(B)** G5A9.

**Figure S3** – XPS survey spectra for **(A)** G5 and **(B)** G5A9.


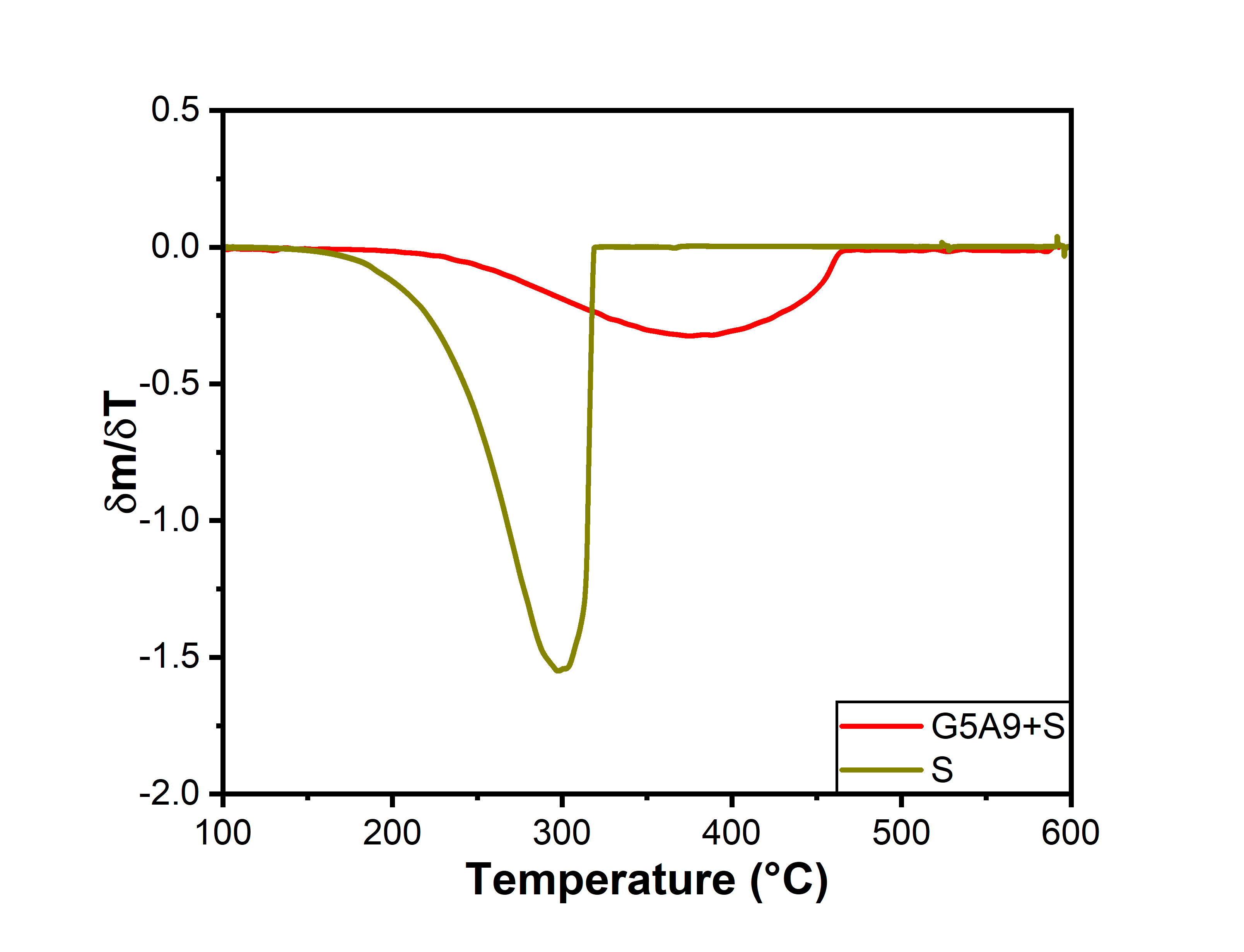


**Figure S4** - Derivate δm/δt vs temperature for G5A9+S (red) and S (olive).


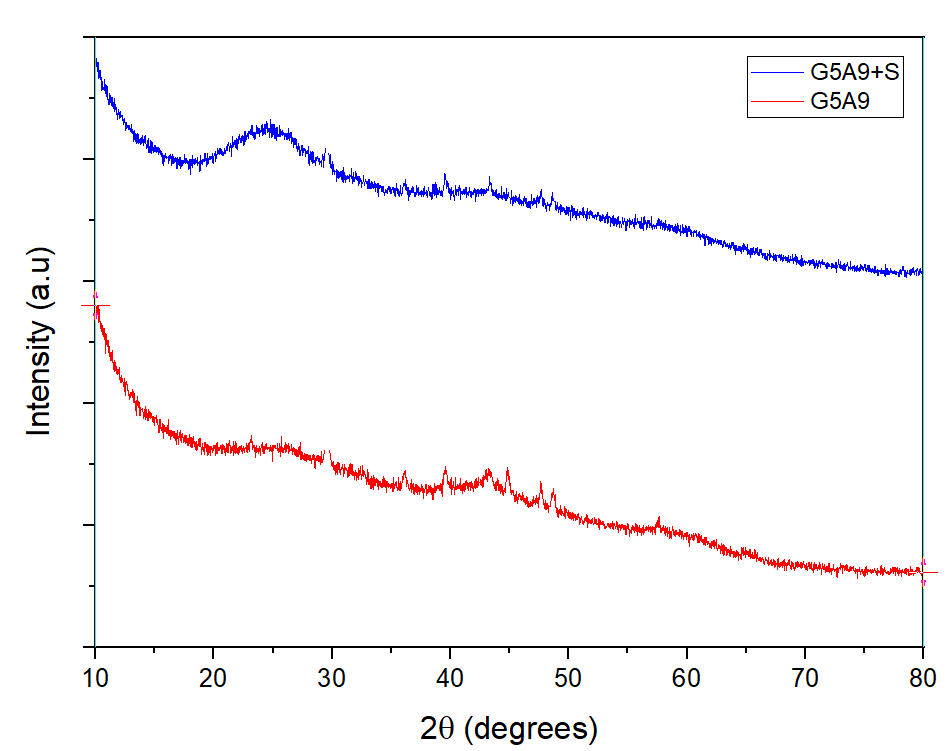


**Figure S5** – DRX for G5A9 and G5A9+S


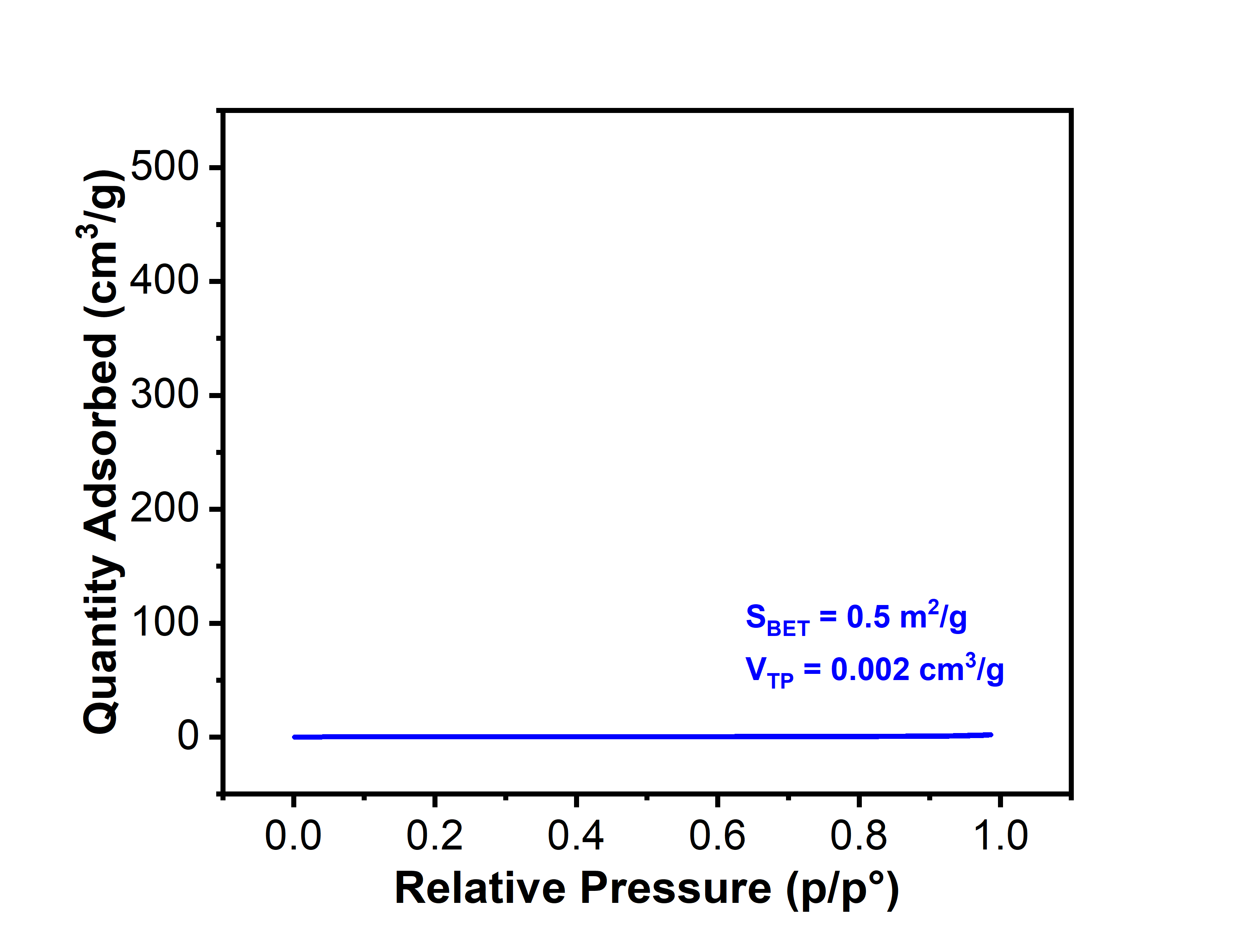


**Figure S6** – N_2_ adsorption–desorption isotherm for G5A9+S


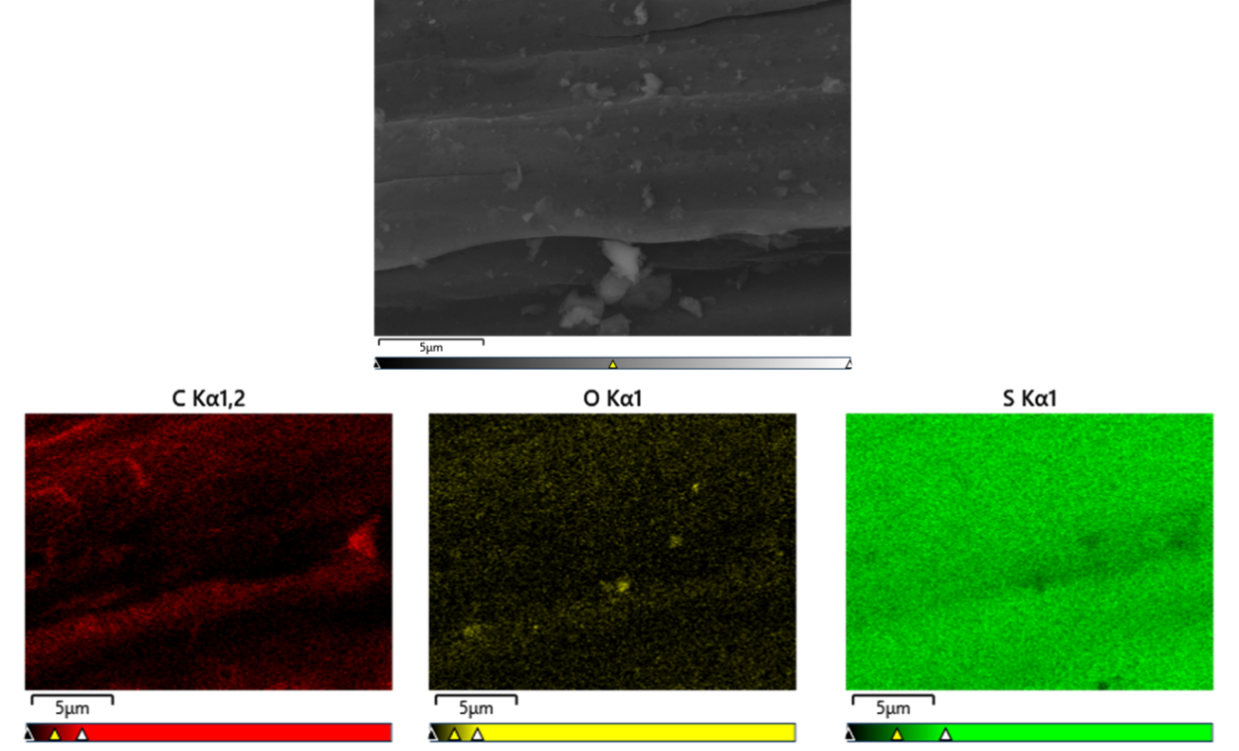


**Figure S7** – EDS mapping for G5A9+S

**Note S1**: Randles–Ševčík equation

The apparent lithium-ion diffusion coefficient (D_app_) was estimated from cyclic voltammetry (CV) data using the Randles–Ševčík equation, based on the linear relationship between the cathodic peak current (i_p_) and the square root of the scan rate (ν^1/2^). The slope obtained from the i_p_ vs. ν^1/2^ plot for the first cathodic peak (~2.2 V vs. Li^+^/Li) was used for the calculation. The diffusion coefficient was determined according to:

$i_{p}=\left( {2.687x10}^{5} \right)ACn^{\frac{3}{2}}v^{\frac{1}{2}}D^{\frac{1}{2}}$ Eq. SE1

where n is the number of electrons involved in the redox process (assumed to be 2 for the first reduction step of sulfur), A is the electrode area (1.13 cm^2^), and C is the effective concentration of the active material (mol/cm^3^). The latter was estimated from the sulfur loading and an assumed electrode thickness.

**Note S2**: Galvanostatic Intermittent Titration Technique

***Overpotential***

In the GITT measurement, the total overpotential (η) can be calculated by the difference between the measured cell voltage (E_meas_) during the current pulse and the voltage at the end of the relaxation period (Eeq), as shown in Figure S5 and Equation SE2 [1]. CCV is the close-circuit voltage and QOCV quasi-open-circuit voltage.

η = |E_meas_ - E_eq_| = |CCV - QOCV| Eq. SE2

Once the overpotentials were obtained, they were plotted as a function of the GITT steps, for charging and discharging.

There were also plotted overpotential vs State of Discharge (SOD), which were calculated by the following equation

$SOC (\%)=\frac{GITT step x 100}{\Sigma_{GITTsteps}}$ Eq. SE3


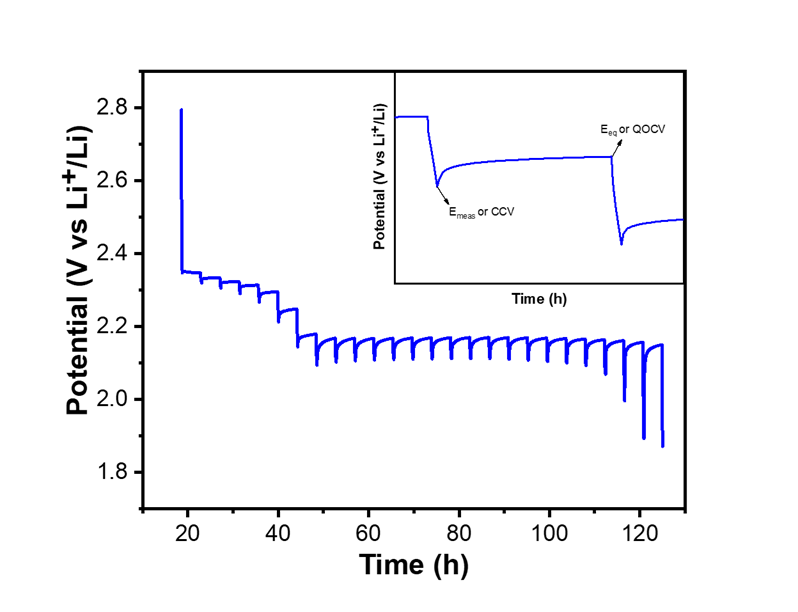
GITT step indicates the number of the step and ΣGITT_steps_ is the sum of all the GITT steps.

**Figure S8** - GITT voltage curve for G5A9+S cell during the discharge.

***Diffusion coefficient***

The diffusion coefficient obtained by GITT was calculated by following the equation SE4, where V es de electrolyte volume in cm^3^, A the electrode area in cm^2^, $\Delta E_{R}$ the voltage difference on relaxation step, that is equal to E_eq_ of Eq. SE2, and $\Delta E_{P}$the voltage difference of the pulse of current (E_meas_ from Eq SE2).

$D=\left( \frac{4}{\tau\pi} \right)\left( \frac{V}{A} \right)^{2}\left( \frac{\Delta E_{R}}{\Delta E_{P}} \right)^{2}$ Eq. SE4


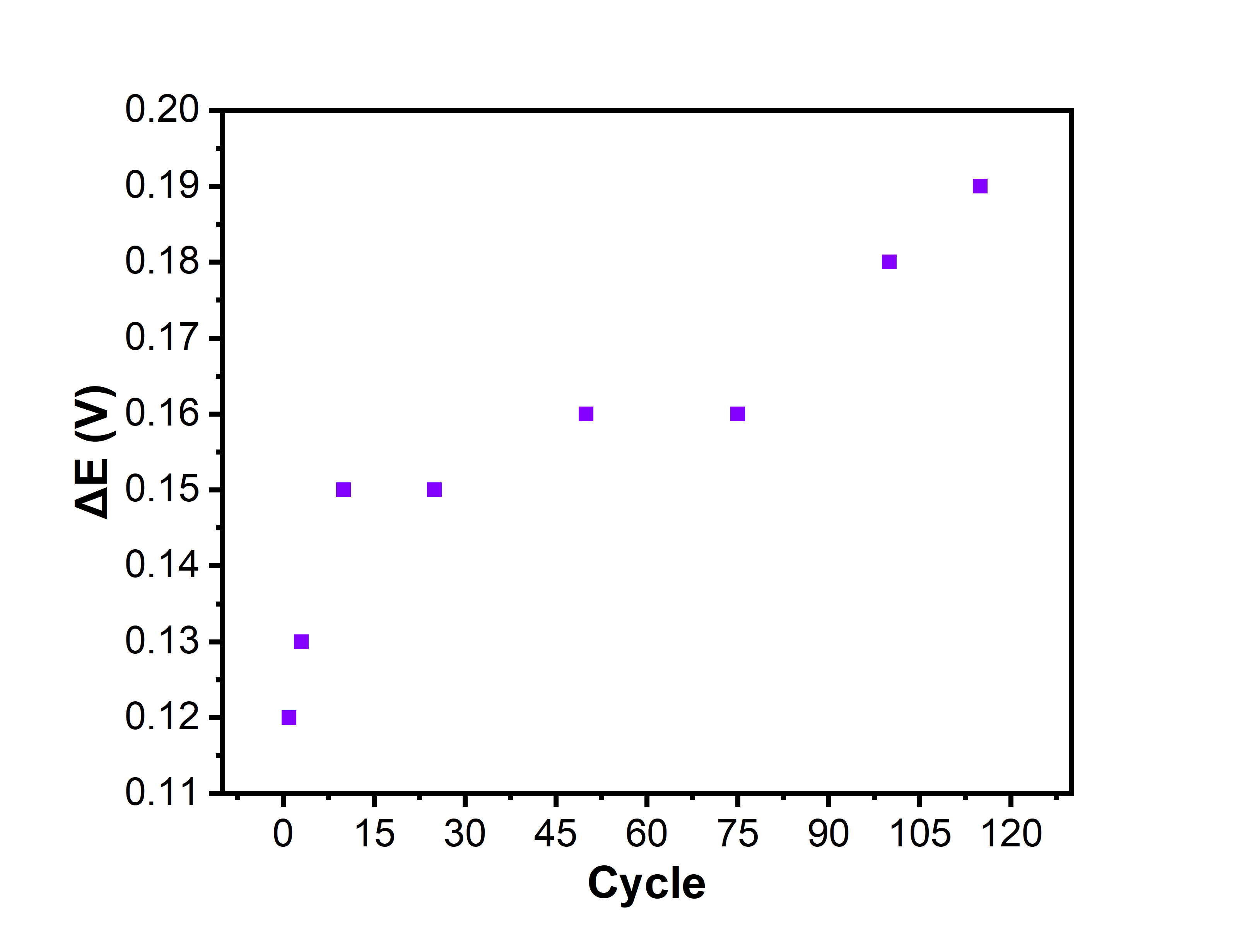


**Figure S9** - ΔE for each cycle for G5A9+S cell. Calculated from Figure 7A.

**Figure S10.** Nyquist plot of electrodes in the fully delithiated state, for the 5th (red) and 10th cycles (blue). For comparison, the Nyquist plot of pre-cycled electrodes is included (black). The measured data (symbol) are presented together with the corresponding fitted curves (solid line).
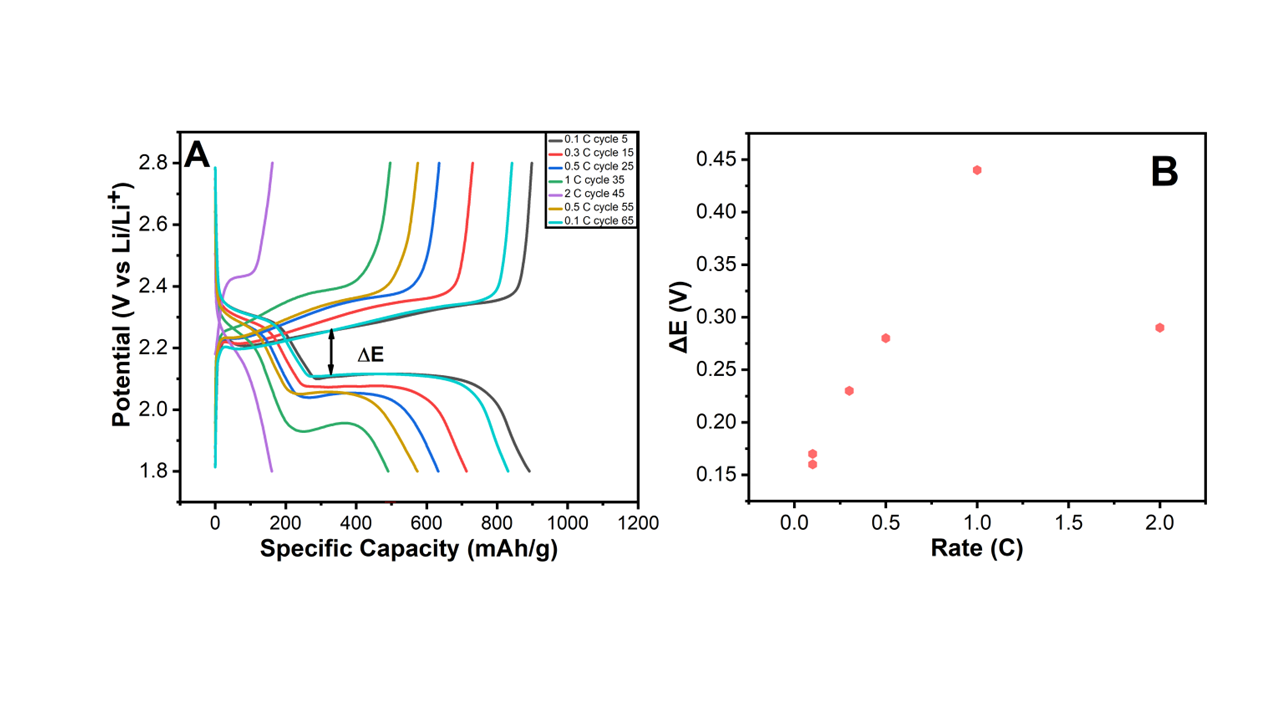


**Figure S11** - G5A9+S charge/discharge profiles of C-rate measurements **(A)** and their corresponding ΔE evolution analysis **(B)**.

**References**

[1] J. Kim, S. Park, S. Hwang, and W.-S. Yoon, ‘Principles and Applications of Galvanostatic Intermittent Titration Technique for Lithium-ion Batteries’, *J. Electrochem. Sci. Technol.*, vol. 13, no. 1, pp. 19–31, Feb. 2022, doi: 10.33961/jecst.2021.00836.
